# Smart Glasses for Facial Recognition and Memory Assistance in Alzheimer's care

**DOI:** 10.1002/alz70858_103276

**Published:** 2025-12-25

**Authors:** Dalene Ephme Ben George, Harshini I, Shivani M

**Affiliations:** ^1^ Sri Sivasubramaniya Nadar College Of Engineering, Chennai, Tamil Nadu, India

## Abstract

**Background:**

Alzheimer's disease is a disorder that causes memory loss, deterioration of cognitive function, and the inability to recognize familiar faces, often leaving patients socially isolated and in emotional distress. To address this, we have developed smart glasses equipped with facial recognition and audio feedback that will provide assistance to the patients in recognizing individuals and recalling relationships. When the system recognizes a face from the database, the individual's name is played along with their relationship to the patient via a connected audio output. Since patients are prone to wandering, we have integrated GPS, allowing the caregivers to monitor the patient's location.

**Method:**

The system utilizes an ESP32‐CAM module for capturing real‐time images and performing facial recognition using pre‐trained facial encodings. The facial data is trained and processed using machine learning libraries like OpenCV, NumPy, and dlib in Python and transferred to the ESP32‐CAM for an embedded operation. Using text‐to‐speech synthesis, the auditory feedback is given to the user through earphones. A touch sensor is used to allow the patients to toggle system features, such as activating or deactivating facial recognition. A GPS module is used for real‐time location tracking, enabling caregivers to monitor the patient's location via a connected mobile application. The system firmware is developed in C, that handles core functions like camera control, communication with touch sensor and GPS and efficient database matching for facial recognition. These can be integrated in power glasses that can be used in daily life.

**Result:**

The recognized faces are matched with a stored database, and the name of the identified person and their relationship is announced to the patient through a speaker or earphones using text‐to‐speech synthesis. This reduces dependency on caregivers and improves the patient's confidence in navigating social and public environments.

**Conclusion:**

Smart glasses help Alzheimer's patients to improve their daily life, along with their social interactions, safety, and the overall quality of life.

